# Research on Chinese Life Cycle-Based Wind Power Plant Environmental Influence Prevention Measures 

**DOI:** 10.3390/ijerph110808508

**Published:** 2014-08-19

**Authors:** Hanxi Wang, Jianling Xu, Yuanyuan Liu, Tian Zhang

**Affiliations:** School of Environment, Northeast Normal University, Changchun 130117, China; E-Mails: wanghanxizs1982@126.com (H.W.); lyyswu@sina.com (Y.L.); zhangt162@nenu.edu.cn (T.Z.)

**Keywords:** wind power, life cycle, ecological destruction, noise pollution, bird migration

## Abstract

The environmental impact of wind power plants over their life cycle is divided into three stages: construction period, operation period and retired period. The impact is mainly reflected in ecological destruction, noise pollution, water pollution and the effect on bird migration. In response to these environmental effects, suggesting reasonable locations, reducing plant footprint, optimizing construction programs, shielding noise, preventing pollution of terrestrial ecosystems, implementing combined optical and acoustical early warning signals, making synthesized use of power generation equipment in the post-retired period and using other specific measures, including methods involving governance and protection efforts to reduce environmental pollution, can be performed to achieve sustainable development.

## 1. Introduction

As a clean and renewable energy source, the level of available global annual wind energy is not only greater than that of hydropower, but also greater than the sum of the available solid and liquid fuel energy forms [1]. Worldwide, the reserves of wind energy in some countries such as the USA, Japan, Canada, Australia, India and China are abundant [2]. At the same time, some countries make better use of wind energy, including Germany, the United States, the United Kingdom and Denmark [3]. In “The Seventh Five-Year Plan” (1986–1990) and “The Ninth Five-Year Plan” (1996–2000), Chinese wind power adopted relatively mature techniques, and industrial capacity development was achieved [4]. Since 1996, global wind power installation has maintained an average annual growth rate above 25%, and wind power has become the fastest growing clean energy source in the World [5,6]. According to Chinese Wind Power Development predictions, by the end of 2020, the country’s total installed wind power will reach 120 million kW, and it will reach 500 million kW by the end of 2050 [7]. In the context of considering the environmental value, wind power generation costs less than thermal power generation [8], so wind power has become one of the most commercial prospects for the development of new energy industries [9] and is thus an important strategic choice for the sustainable development of electricity [10]. 

Currently, wind energy has the most potential for renewable energy development, and Asia is expected to become the largest wind power market. With the development of Chinese industrialization, the current wind design, installation and management techniques are close to advanced world levels [11]. However China still lags compared to foreign countries in terms of technical level, market size and construction, among other areas [12]. Wind power technology is divided into large-scale wind power generation technology and small-scale wind power generation technology [13]. Due to current national policy-oriented reasons, in China, the small-scale wind power industry is limited, whereas the large-scale wind power industry has been developing rapidly [14]. Currently, the Chinese onshore wind power industry is relatively mature, but the development of offshore wind energy has just started and still lags compared with the mature foreign offshore wind industries of other countries [15,16,17]. China should enhance the level of the independent research and development, manufacturing technologies, core components, management experience, equipment testing and other aspects to facilitate risk assessment and avoid construction risk according to local conditions. Only in this way can we achieve sustainable and healthy wind power development [18,19]. When developing a sustainable energy strategy, the impact of wind power development on the environment should not be ignored [20]. Studies on the environmental impact of wind power plants, performed by many foreign and domestic experts and scholars, mostly focus on a particular period, a particular aspect or a particular environmental factor [5,8,10,12,13,14,15,16,17,18,20,21], but no one has studied the environmental impact of the entire life cycle of wind power plants. 

In-depth study of wind power plant environmental impact protective technologies throughout their life cycle (including the construction, operation and retired period) should allow the introduction of new technologies and effective measures to reduce their environmental impact, which has positive significance and shall play an important role in the benign development, construction and operation of wind power projects.

## 2. Material and Methods

**Figure 1:**
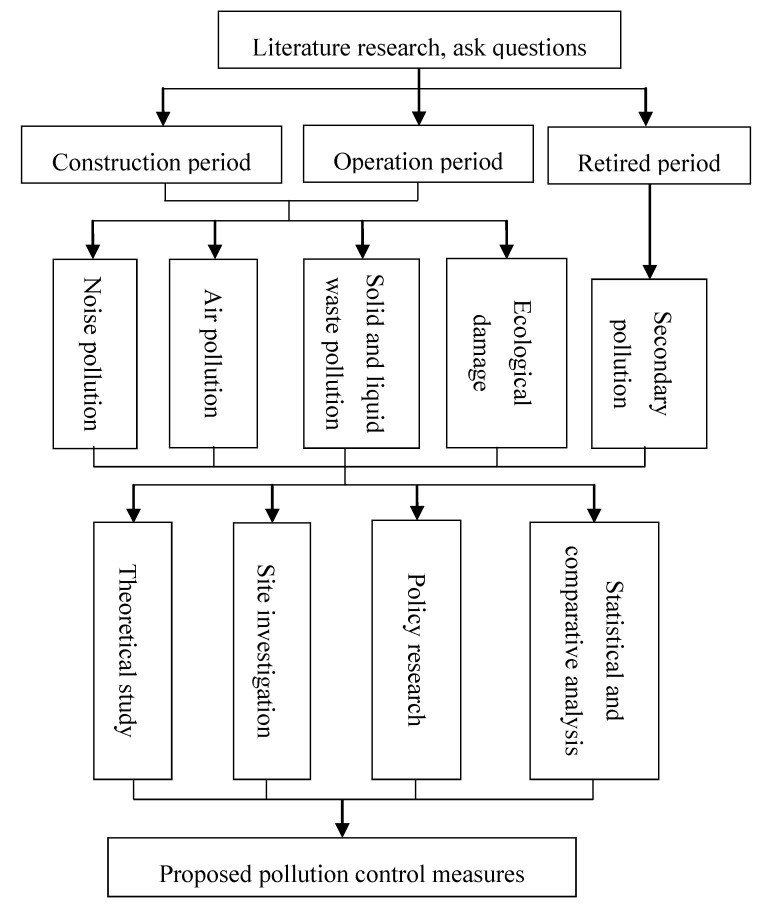
Research Procedures and Methods.

Based on literature research, we used a combined method involving theoretical analysis and field survey that took into account full knowledge of the status of the Chinese wind power industry and national policy to explore the prevention of technical problems involved in the environmental impact of wind power plants throughout their life cycle. The flow of the main research methods and procedures is shown in Figure 1.

### 2.1. Literature Review

On the basis of an analysis of domestic and international literature and engineering data, combined with Chinese national conditions, technical requirements and methods suitable for Chinese economic development were identified.

### 2.2. Scene Investigation Methods

In the analysis of projects constructed and operated in China, the focus was on the identification of environmental issues during the construction and operation periods, as well as ecological restoration and resource utilization after retirement.

### 2.3. Study Combination of Theory and Practice 

Based on the related theory analysis and comparison with actual projects, a combination of theory and practice was achieved.

### 2.4. Policy Research

In the context of maintaining national standards compliance, foreign and domestic related technology policies for environmental protection, and taking into account the current level of wind power development, proposals to improve environmental protection technology were developed. 

### 2.5. Statistical and Comparative Analysis

Statistical methods were used to analyze the relevance and legitimacy of the currently available data. The focusing was on comparing and analyzing different wind power plant projects to identify the relevant factors that affect the environment and provide a basis for environmental technology improvements.

## 3. Environmental Impact Analyses of Wind Power Plants

### 3.1. Construction Period

Wind power generation projects require a large amount of land resources, especially temporary coverings. In addition, construction worker and machinery activities have a great impact on the environment. To reduce the environmental impact of construction and the difficulty and cost of later government, environmental impact analysis is necessary.

#### 3.1.1. Noise Pollution

A major source of noise from wind power projects during the construction period is the noise of construction machinery, in particular that from construction, equipment installation and construction and haulage vehicle operations. Mechanical noises produced by these sectors have a certain impact on the surrounding environment. In particular, for some noise-sensitive animals, the noise will not only affect their normal day-to-day living, but it can also have a negative impact on their procreation.

#### 3.1.2. Air Pollution

During the construction period, air pollution includes dust produced by passing vehicles and machinery construction, exhaust emitted by construction machineries and vehicular traffic and harmful ammonia gases released by the concrete after antifreeze addition during winter construction [21]. Currently, winter fog and haze also occur in China, so air pollution cannot be ignored.

#### 3.1.3. Wastewater, Solid Waste and Waste-Contaminated Mud Pollution

The construction waste of wind power projects comes from wastewater, garbage generated during the construction process and packaging of workers’ wind power equipment, in addition to waste directly produced by the workers working and temporary living in the area. This wastewater and solid waste can not only affect the landscape, but also can pollute groundwater after rainfall. In particular, garbage eaten by birds after decay can cause disease epidemics. Waste mud is generated by the pile foundation and construction. Given the particularities of a requirement for 360° withstanding of repeated loads and large eccentric direction of the force during the operation of wind turbines, most projects use pile foundations [22]. The process of preparing a pile foundation and construction will produce waste mud. If improperly handled, this waste mud will not only pollute the groundwater, but it will also cause ecological damage, especially after the addition of soda to waste mud, which can, if severe, destroy the soil structure and cause desertification.

#### 3.1.4. Ecological Environment Destruction 

Plant roads, fan bases, booster stations, transmission lines and other occupied spaces in wind power projects will damage vegetation, reduce biomass, decrease species diversity and produce landscape fragmentation and other ecological environmental effects [23]. During the construction process, because the area of temporary space occupied is larger than the permanent occupation area, there is destruction of plants and soil pollution. In Qinghai [24], Yancheng [23], Inner Mongolia, Jiuquan Gansu [25], Changtu Liaoning [26] and other locations in China, large wind power construction areas has caused some difficulties with restoration of the ecological environment, as mentioned in Table 1.

**Table 1:** Land Area Occupied during Wind Power Construction.

No.	Project Title		Temporary Cover (hm^2^)	Total Cover (hm^2^)	Proportion of Temporary Cover (%)
1	Wind Power of Changtu Liaoning	Generators	--	1.55	--
		Collector Lines	--	0.09	--
		Roads	--	12.05	--
		Booster Stations	--	14.24	--
2	Wind Power of Huitengliang Xilin Gol League in Inner Mongolia	Datang of the Sixth Wind Power Projects	69.95	138.48	50.51
		China Guangdong Nuclear Power 300 MW Wind Power Concession projects	85.79	139.98	61.29
		Sinohydro First Wind Power Project	22.54	31.12	72.43
3	Wind Power of Yancheng Jiangsu	Dafeng First Project	17.37	23.70	73.29
		Dafeng Second Project	10.55	41.60	25.32
4	Wind Power of Jiuquan Gansu	Guazhou	1684.45	3357.80	50.17
		Yumen	684.75	1236.85	55.34
		Mazong Mountain	551.53	1068.08	51.64

### 3.2. Operation Period

The effects on the environment of wind power during the operation period mainly stem from the noise of the wind power equipment, influence of fan blades on birds, electromagnetic radiation produced by the transmission lines, wastewater and garbage produced by the people who are on duty, light and the light pollution produced by the fan blade, oil and solid waste produced by equipment maintenance and ecological damage related to the repair of vehicles.

#### 3.2.1. Impact of Electromagnetic Radiation

The intensity of the electromagnetic field in the local space or the whole space can be so large that it results in harm to organisms in the environment. In particular, the thermal and non-thermal effects caused on the human body by electromagnetic radiation can cause headaches, neurasthenia, reproductive organ damage and other pathological reactions [27]. The electromagnetic radiation can have a greater influence on breeding females and the chicks of rare passing birds. Underwater cables related to the wind power installations in the sea cross the ocean, result in fish suffering from the magnetic field effects. In particular, the survival rates of spawned fish are lower during the spring breeding season.

#### 3.2.2. Noise Effects

The sources of wind power generation noise during the operation period are mechanical noise and aerodynamic noise [28,29], and they are a function of wind speed [30,31]. For the currently built projects, when monitoring noise we can consult the documents “The Measurement Technology for Wind Turbine Acoustic Noise” [32] released by the International Electrotechnical Commission (IEC) and “Noise Limits and Measurement of Wind Power Plants” [33] produced by the Chinese government. Because the influence of the noise is long-term, we should consider prediction methods to understand the extent of the influence on the surrounding residents, the duty-room workers and rare animals.

Wang *et al*. [34] others measured 10 sets of three wind farms, and the monitoring results for the measured noise of the wind fans along the coast in Shandong Province are shown in Table 2. The monitoring results indicate that the level of the noise is not proportional to the capacity of a single wind farm set, but a reduction of the monitoring distance can be observed.

**Table 2:** Monitoring results for wind farm noise for power plants along the coast in Shandong province.

Fan Position		Distance between the Monitoring Point and Fan’s Seat (m)	Wind Speed on the Ground (m/s)	Load (kW)	Measured Value (dB(A))		
					Noise of the Falling Fan Blades	Noise of the Rising Fan Blades	Average
CDFS Wind power plant **(**600 kW**)**	2^#^ unit	10	3.6~5.2	300~580	58.4~63.5	56.7~61.7	57.6~62.7
	3^#^ unit	10	1.6~4.5	50~470	52.3~63.1	50.6~61.5	51.5~62.4
RCGH Wind power plant **(**1250 kW**)**	14^#^ unit	30	3.0~6.0	330~1250	63.3~67.4	65.5~68.0	64.7~67.7
	15^#^ unit	30	3.0~6.0	330~1250	64.8~66.0	65.0~67.0	65.2~66.5
	33^#^ unit	30	3.0~6.0	330~1250	66.0~66.7	65.5~67.7	65.9~67.2
RCHN Wind power plant **(**1500 kW**)**	5^#^ unit	35	3.0~6.0	300~1500	49.6~55.6	49.4~56.3	49.5~56.0
	12^#^ unit	35	3.0~6.0	300~1360	51.9~57.8	52.0~57.1	52.0~57.5
	13^#^ unit	35	3.0~6.0	300~1360	51.5~58.0	51.6~58.3	51.6~58.1
	25^#^ unit	35	3.0~6.0	300~1360	49.6~56.6	49.7~56.9	49.7~56.5
	32^#^ unit	35	3.0~6.0	300~1360	49.2~55.1	49.3~55.6	49.3~55.4

Fu [26] has monitored and made predictions about the wind power project in Changtu County, finding that for attenuation of the noise, Equation (1) should be used, and the superposition of the noise value should be determined using Equation (2). The distance between the predicted point and the sound source is 230 m, and we analyzed the monitoring results comparatively by choosing eight different places at different times of day and night using Equations (3) and (4) for the calculations. The statistical analysis and deviation results are shown in Table 3:

*L_r_* = *L*_0_ − 20log*r*/*r*_0_ (1)

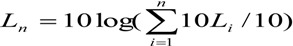
(2)
where *L_r_* is the sound pressure level of the predicted point in dB(A); *L_0_* is the sound pressure level away from *r_0_*, in dB(A); *r* is the distance between the predicted point and the sound source measured in meters; *r_0_* is the distance between the sound source and the point where the level of sound is measured in meters; *L_n_* is the synthesis of the n source sound pressure level in dB(A); *L_i_* is the value of the different sound pressure in dB(A):

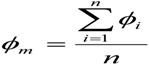
(3)

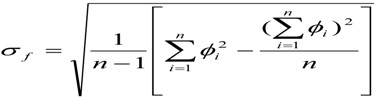
(4)
where *ϕ_m_* is the average and *σ_f_* the standard deviation.

Zhai *et al*. using an improved version of the NASA wind power noise model, compared predicted and measured results. The model is a semi-empirical model, which was developed by NASA in the USA. It also allows prediction using a far-field axial noise model of double bladed wind turbines that have an obtuse trailing edge [35]. The main calculation formulas of the improved NASA model are as follows:

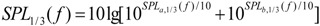
(5)

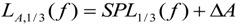
(6)

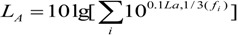
(7)

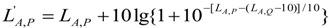
(8)

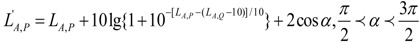
(9)
where *SPL_a_*_,*1/3*_ is one-third of the octave band sound pressure level of the turbulence noise; *SPL_b_*_,*1/3*_ is one-third of the octave band sound pressure level of the turbulence boundary layer noise; *L_A_* is the total A sound pressure level from superimposing each one-third frequency of the A sound pressure level; *L_A_*_,*P*_ is the unmodified A sound pressure level at point P; *L'_A_*_,*P*_ is the revised A sound pressure level at point P; *L_A_*_,*Q*_ is the unmodified A sound pressure level at point Q when the distance is d meters away from tower footing from the direction of α = 0°. 

**Table 3:** Predicted results of statistical analysis of the monitored noise in residential areas.

Project		Measured Values (dB)	Predicted Values (dB)	Difference between the Measured Value and the Predicted Value (dB)		Predicted Deviation (%)	
				Range	Average	Range	Average
1^#^~8^# ^unit	Day	35.2~41.6	43.5~45.2	3.6~8.3	5.1	8.7~23.6	13.3
	Night	32.3~36.1	43.2~43.7	7.6~10.9	9.4	21.1~33.7	27.8
Standard deviation		--	--	1.6/1.06		5.1/4.1	

As seen from Table 3 and Table 4, the deviation and the standard deviation obtained using the improved NASA model for prediction are smaller than those obtained using the traditional methods as represented by Equations (1) and (2). The accuracy rate of the method is very high, so we can use the improved NASA model to make predictions when analyzing the impact of the operation of wind power on surrounding sensitive points. 

**Table 4:** Results of the comparison between the predicted value and the measured value.

Project		Measured Value (dB)	Predicted Value (dB)	Difference between the Measured Value and the Predicted Value (dB)		Predicted Deviation (%)	
				Range	Average	Range	Average
Unit	1^#^	43.2~54.6	43.5~52.1	−0.6~2.5	0.2	−1.3~4.6	0.4
	2^#^	51.0~55.9	50.8~52.7	−0.7~3.5	0.8	−1.4~6.3	1.3
	3^#^	45.4~51.0	47.0~50.5	−1.6~0.5	−0.6	−3.5~9.8	0.8
Standard deviation		--	--	1.4		3.8	

#### 3.2.3. Wastewater, Solid Waste and Oil Pollution

During the operation period, the wastewater, solid waste and garbage produced by duty-room workers and also the oil pollution produced by equipment maintenance can pollute the environment. Wastewater, solid waste and oil pollution must be handled properly given their long-term effects and persistence, even though the relative production level of such pollutants is low at wind power plants.

#### 3.2.4. Light and Light Pollution

Because the vertical rod and fan blades are coated with white paint, reflective light can create light pollution that affects nearby residents. In addition, the light behind the fan will have a greater influence on vegetation. 

#### 3.2.5. The Impact of Wind Farms on Ecology

During the operation period, the impact of wind farms on the local ecosystem mainly involves ground pollution by oil generated during equipment maintenance and repair, rolling damage to ground vegetation from vehicular traffic and harm to birds from fan blades. In the past 20 years, wind power has made considerable progress but also has had a negative impact on birds [35,36]. As the wind turbines produce noise during operation, the habitats of birds have changed [37,38,39]. The footprint of wind farms directly occupied by wind turbines is approximately 2% to 5% [40]. Because of the risks of bird casualties during collision with the blades when they are flying in wind farms, the construction of wind farms has changed bird habitats and foraging sites [41]. The impact of the construction of wind farms on birds includes the following four aspects:

(1) The construction of wind farms reduces bird habitats
  In Nysted, Denmark, wind farms monitor the behavior of birds through radars. The results indicate that the lengths of the periods where daytime-acting Anatidae enter their original habitats in the wind farms are rapidly decreasing [42]. The reduction of birds' habitats, in particular the reduction of living space, affects bird breeding and survival.
(2) The construction of wind power changes the migratory paths of birds
  Monitoring of bird migration by Nysted offshore wind farms in Denmark is also conducted using radars. The results indicate that the birds bypass wind farms by as much as 3000 m during the day and as much as 1000 m at night [37,38,39]. Studies have demonstrated that water birds begin to make way for wind farms at a distance of 100~3000 m [43]. There are also studies demonstrating that, at the Horns Rev and Nysted offshore wind farms, construction of wind farms is a major factor affecting the flight paths of *Melanitta nigra* [43,44]. Avoiding wind farms increases energy consumption for birds during migration, which is very unfavorable to the survival and reproduction of birds.
(3) Construction of wind power impacts the survival of birds
  Migratory birds can crash into or be injured by operating fan blades, which results in the most serious impact on birds from wind farms. Studies have demonstrated that passerine birds suffer the largest number of bird hitting fan accidents, and they account for 80% of birds killed by crashes [45], whereas only 2.7% of such birds are prey birds [46]. Endangered birds being killed by impacts have received the greatest amount of attention [47]. These deaths have a significant effect on the survival and recovery of bird species.
(4) Construction of wind power has an impact on breeding birds
  The impacts of constructing wind farms on birds occur during the migration period, habitat usage period and breeding period. The impact to birds during these three stages is mainly caused by the death of birds from colliding with fans during migratory fighting and occupation of habitats by birds during the reproductive period and winter [48,49]. Staff working on wind farms and tourists can also influence nearby birds to a certain extent. In particular, breeding bird nesting success rates decrease [50].
  The above four wind farm factors that affect birds are interrelated and occur simultaneously in many cases. Therefore, the impact of constructing wind farms on birds cannot be ignored, especially for endangered birds.

### 3.3. Retired Period 

Though existing wind farms can conflict with plans, and the operating efficiency of wind power equipment can be reduced by climate change, as well as with difficulties with regional grid and other special reasons, wind farms generally do not have problems once retired. Here, retired refers to the decommissioning of wind power equipment. Wind power equipment that becomes obsolete, developed low operating efficiency or improper function will face retirement. Chinese wind power plants are in the development stage, and the current early wind power plant equipment is facing retirement issues. Therefore, proper handling of wind power equipment after retirement and decommissioning of wind turbines to aid in their finding a second life is an important aspect of achieving sustainable wind power plant development.

## 4. Chinese Wind Power Plant Environmental Influence Prevention Measures

### 4.1. Construction Period

Wind power plants have some influence on the environment during their construction period, including noise pollution, air pollution and water pollution caused by solid wastes and waste mud, in addition to ecological destruction in which waste mud pollution plays a dominant role. It is the belief of the authors that when considering the environmental effects and engineering aspects, it is better to combine traditional methods and new technology together.

#### 4.1.1. Traditional Technological Measures

(1) Noise Pollution
  To reduce mechanical noise pollution during the construction period, the main measures adopted are as follows: ① Location avoidance ‒ avoidance of building the wind plant in an area where humans live and where there are animals sensitive to noise; ② Construction period control—avoid working while animals are resting; ③ Use of advanced machines and equipment to reduce noise; ④ Optimization of the construction scheme and accelerated construction progress.
(2) Air Pollution
  Some measures to reduce air pollution are follows: ① Optimize the construction scheme and reduce periods of active vehicle traffic; ② Sprinkle water on the construction roads on a regular basis to prevent dust floating; ③ Avoid construction in the cold seasons to reduce the use of antifreeze.
(3) Wastewater, Solid Waste and Waste Mud Pollution
  Some of the main measures to reduce solid waste pollution are as follows: ① Optimize the construction scheme to reduce the production of wastewater and solid waste; ②Set up special wastewater and garbage recovery processes and transport the wastewater and garbage out of the wind plant area after collection. As for the waste mud produced by pile foundation construction, the adopted measures include the following: ① Choose advanced equipment to reduce the waste mud production, such as selecting advanced rotary drilling rigs instead of the traditional direct and reverse circulation drilling rigs; ② Set up waste mud pits in the construction area, and then transport the muddy wastes out the area and dispose of them after completion of construction.
(4) Ecological Destruction
  Some of the main measures to reduce the influence of wind plant construction on the ecosystem are as follows: ① Optimize the construction site ‒ select an area where there is less biomass to increase the land use efficiency; ② Implement ecological restoration and compensation in time; ③ Control the width and length of the construction road by optimizing the construction scheme; ④ Strengthen the education of construction personnel and establish a system of rewards and punishments; ⑤ Keep the topsoil alone for backfill to restore soil rationally based on excavation, cable channels and so on. For temporary topsoil yards, temporary prevention measures should be adopted, such as solid bag protection, pat solid and surface covering with straw mattresses, film covering fiber cloth, *etc*. [51]; ⑥ From the perspective of government management, strengthen the supervision of ecological environmental supervision.

#### 4.1.2. New Technology Measures

Ecological destruction and waste mud pollution are the main influences wind plants have on the environment during the construction period, and they are also research focuses. A positive and effective way to reduce ecological destruction is optimization of the construction scheme to reduce floor area and reduction of the required construction period by system analysis. The best method to reduce waste mud pollution is efficient resource utilization.

**Figure 2:**
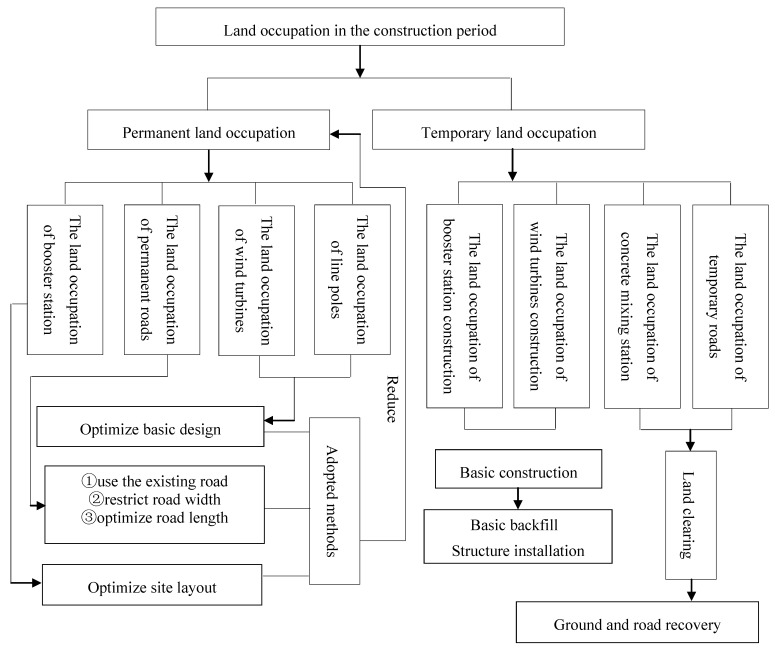
System analysis figure of land occupation during the construction period.

**Figure 3:**
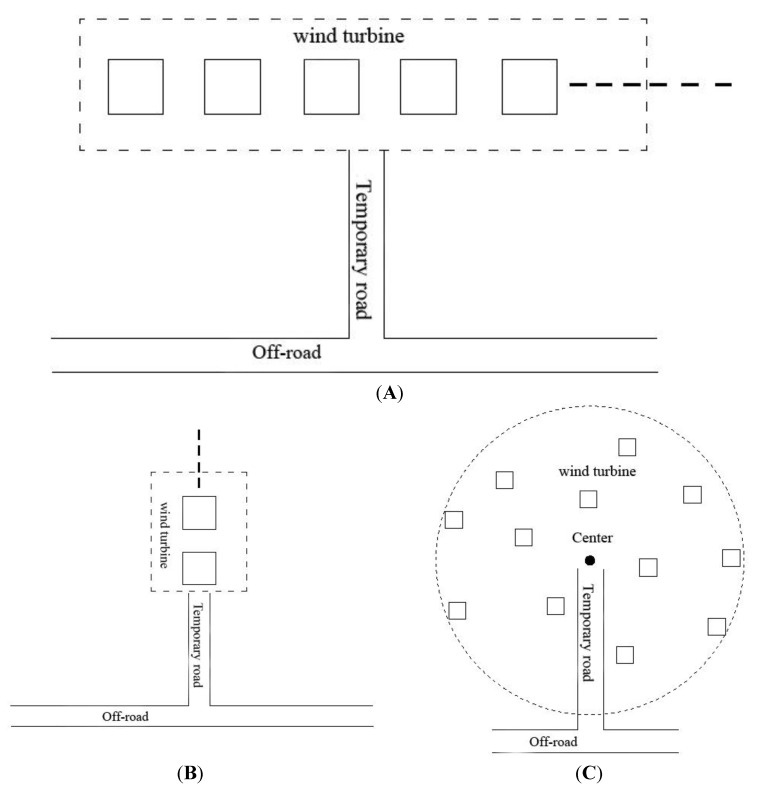
Optimal placement of the wind turbines lines (**A**) Parallel with the road, (**B**) The form of vertical road and (**C**) The form of approximate circular.

As shown in Figure 2, system analysis has been used to optimize the construction scheme to reduce the noise pollution, air pollution, production of solid wastes and ecological destruction during the construction period. The issues requiring attention during construction mainly include the following:

(1) When restricting the road width, set the wind turbines parallel or vertical with the off-roads, as well as establish a layout of the wind turbines approaching a circle to optimize temporary road construction, as shown concretely in Figure 3.
(2) Construction workers living area should be placed in nearby villages.
(3) Ensure that site supply times are no longer than the initial concrete setting time and attempt to set up the concrete mixing stations in the nearby villages as to avoid occupying the construction land.
(4) Control the time interval between foundation construction and installation, adopt flow production and shorten the construction period.
(5) Build the wind turbines, booster stations and line poles far away from ecologically sensitive areas, and choose locations near roads and where there is less biomass as much as possible.

**Figure 4:**
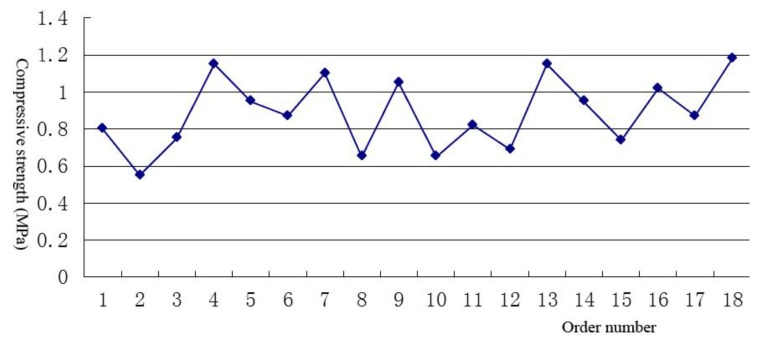
Compressive strength test results for a hardened layer of an antifouling structure.

To solve the environmental problem of ground pollution by waste mud at the construction site, the authors have patented a protective layer structure to prevent ground pollution (The Chinese patent application number is 201310688451.4). The patent uses a floor contamination protection structure that mainly consists of waste mud, soil or sand materials, in addition to plastic film to reduce waste mud, and forms an impermeable layer. The antifouling structure mainly includes a hardened layer, protective cushion layer and impermeable layer. The hardened layer can bear the upper drill or pile foundation load, and the protective cushion layer can prevent the impermeable layer from being destroyed. In addition, the impermeable layer can prevent the waste mud sprayed by the pile foundation or the drilling rig from leaking into the solid layer and causing ground pollution. The hardened layer thickness is 200~500 mm. The preparation method for the layer involves mixing cement (10%~20%), sodium silicate (0.5%~2%), fly ashes (0.5%~1%), slaked lime (1%~2.0%) and precipitation slurry (75%~88%) and stirring them evenly. The impervious layer uses PE plastic films with a thickness of 0.01~0.25 mm. The protective cushion layer is just soil or sand from the construction site, and its thickness is 50~100 mm. The method was used to good effect during the construction of the Jilin Qian’an wind plant. Samples from the hardened layer were tested (150 mm × 150 mm), and the results are shown in Figure 4. The results of the calculation of the compressive strength of the 18 sample results are shown in Table 5.

During the construction process, approximately 2039 m^3^ of waste mud was produced. If it were all to be transported out, with each truck loading 15 m^3^ of waste mud then eight trucks are required to make seventeen trips. Assuming the site is 55 km from the city waste mud disposal area, the transportation charges will be very expensive. Using this patented method, 1926 m^3^ of waste mud could be processed and all of the waste mud used for surface hardening after processing. Compared with the method involving outward transportation of waste mud, the patented method reduces cost by 26.7%. In addition, the cost of the non-patented method will be even higher when the cost of ecological restoration is considered. Practice indicates that the method can effectively solve waste mud pollution problems and also save on engineering costs.

**Table 5:** Statistical evaluation of the compressive strength test results for the hardened layer of an antifouling structure.

Name	Strength Range/MPa	Average/MPa	Standard Deviation/MPa	Coefficient of Variation	Standard Value/MPa
Result	0.55~1.18	0.89	0.19	0.218	0.89 ± 0.08

### 4.2. The Operation Period

The environmental impacts of the operation period mainly include electromagnetic radiation, noise, water, solid waste, oil, light, and light shadow pollution and ecological damage. Among these, noise pollution and ecological damage have the main effect on birds. Taking advantage of engineering aspects in attempts to reduce these issues by combining traditional methods and new techniques, as based on full consideration of current environmental protection requirements, we obtained good results.

#### 4.2.1. Traditional Technical Measures

(1) Electromagnetic radiation pollution
  The radiation impact of a wind power plant mainly comes from the transmission lines. The wind power protective measures on the land mainly include the following suggestions: ① set warning signs, and control the distance between transmission lines and human activity; ② control insulator surface discharges; ③ reduce the probability of producing sparks because of poor contact [52]; ④ strengthen operations and maintenance thus ensuring stable operations. With regard to offshore wind power, to prevent fish from being impacted by electromagnetic radiation, we must first consider maintenance of operational stability, decreasing magnetic inductive intensity, and implementing quarantine measures if necessary.
(2) Noise pollution
  The main measures to prevent the noise of wind power plant are as follows: ① for new wind power plants, the measures are site selection avoidance, optimizing site layout and setting reasonable noise protective distances; ② for established wind power plants, the measures are establishing suitable plants and placement of noise barriers; ③ maintain wind turbines at a good operation level; ④define the noise affected-zone, and forbid construction of new residential areas there; ⑤ consider moving the affected residents if there are a small number.
(3) Wastewater, solid-waste, and oil treatment
  Treatment measures for domestic wastewater, biological waste, solid waste and oil on-site include the following: ① use a specialized wastewater collection pool and garbage recycling pool; ② clean up solid waste produced by repairs in a timely matter time; ③ clean up surface oil, pack it with bags or buckets, and ship offsite. The domestic wastewater produced during the operation period should be discharged into an anti-permeability storage system, cleaned up and transported periodically, as it can serve as additional fertilizer with manure.
(4) Light and light shadow pollution
  The protective measures for light and light shadow pollution include the following: ① during the planning period, perform protection distance calculations, and within that distance, forbid the construction of new residential buildings [53]; ② for residents within the zone affected by light, a light barrier can be set up; ③ for the established residential areas in the shadow zone, we can consider moving people who are severely affected.
(5) Ecological impacts
  The protective measures during the operation period concerning ecological damage include the following: ① try not to use vehicles when hauling, and if it is necessary use scheduled routes; ② for materials that need to be removed or repaired on-site, the ground surface should be covered by sheet or plastic film; ③ restore destroyed vegetation in a timely manner after maintenance; ④ for ecological-sensitive zones, set up fences and forbid crossing of the zones without permission. During the construction of wind power plants, the main measures adopted to reduce the influence on birds are as follows:
  ① Avoid in site selection during the planning period. Site selection of wind power plants should involve avoiding high-density region of bird life, especially the migratory passages and waterfowl districts. To protect endangered birds, areas containing their breeding grounds, feeding grounds, wintering grounds, habitat, courtship sites and courtship displays in the air [36] should be off-limits for wind power plants. In addition, large area continuous bird habitat divisions should be avoided [54].
  ② The main measures considered during design are as follows: (1) taking into account the terrain conditions, place wind turbines in groups, their orientation should be parallel to the bird migration direction, and adjacent wind turbines should leave a wide enough flight corridor; (2) for wind power plants located near migratory passages, the wind turbine should not have red flash or sodium vapor lamps, as these disturb the migration of birds [36].
  ③ Paint wind turbine blades and transmission lines with a cautionary color as birds’ judge obstacles on flight paths by sight when flying, and cautionary colors will help reduce the possibility of bird collisions with wind turbine blades and transmission lines, as the color will facilitate evasion by birds and decrease the probability of collision. Successful Japanese experiments indicate most birds have a high sensitivity to orange-red and white, which can be used as cautionary colors [55].
  ④ Establish a bird repellant system. For established wind power plants, or when it is inevitable that birds will be affected after comprehensive consideration, a bird repellant system should be established. Based on airport bird collision prevention experiences, the use of dummies and threatening eye designs can repel birds. In addition, the broadcasting of the sound of birds’ predators and electronic cannons can repel birds. These bird repelling methods can be combined in a “three-dimensional gradient airport anti-bird system” [36,56,57].

#### 4.2.2. New Techniques and Measurement

The environmental impact during the wind power plant operation time is mainly the result of noise and ecological destruction affecting birds. On the basis of the current investigation into Chinese wind power plants, noise pollution predominantly occurs with plants built during the early stages of development, and most plants have since taken corresponding measures. New wind power plants mostly avoid this issue via planning and site selection. Thus, reducing wind power construction injury to birds is the key measure in the new development period.

To solve this problem, the authors have applied the invention “photoelectric sound wave joint warning sign bird repelling devices.” The design principle for the device uses birds’ sensitivity to light and sound, by loading the warning signs onto the blades or pylons of wind equipment, such that when birds pass by, they receive the light signals or acoustic warning signs, and then, they consequently choose to avoid the wind plant, which thus successfully would achieve the goal of repelling birds. The device mainly uses acoustic warning during daytime and light signals at night, which can prevent the interference of external conditions. In addition, the effect is obvious after application. Tests have proven that the technology does not pollute the environment. The invention has low power consumption, using only two 1.5 V batteries. The device can normally operate for around one month. Therefore, the technology has potential for popularization and application.

### 4.3. Retirement Period

At present, wind turbines commonly use thermosetting resin matrix composite materials, and mostly involving stacking methods, which requires massive land resources [58]. Because of the situation in China, the country has mainly adopted the physical recycling method, chemistry recycling methods and energy recovery [59]. The physical recycling method involves the use of created waste as a raw material after crushing or melting. Chemistry recycling methods include pyrolysis, super-critical fluid method and the dissolution method. Energy recovery involves burning the waste that contains sources of organics or entirely organics and converting that heat energy into other energies. By synergistically utilizing these three method types, we can use energy effectively.

As chemistry recycling methods and energy recovery can generate secondary pollution, they are rarely used. In accordance with China's current national conditions, the physical recycling method is primarily used and only supplemented with the chemistry recycling methods and energy recovery.

## 5. Conclusions

Our research on the environmental impact on the life cycle of a wind power plant indicates that in addition to traditional methods, we should place an emphasis on developing new technology to effectively reduce the environmental impact of these types of plants. According to research, the suggested preventive measures to reduce a plant’s environmental impact include the following: ① optimize the construction scheme, and reduce noise pollution, air pollution, discharge of solid waste and ecological damage during the construction period; ② use a protective layer structure that prevents ground contamination to effectively minimize waste mud’s effect on the environment during foundation construction; ③ adopt a combination of prediction and actual monitoring measures to deal with the noise during the operation period; ④ set up photoelectric and sound wave joint warning sign bird repelling devices to reduce bird injury; ⑤ fully consider the material characteristics of retired equipment for potential recycling and reuse.
